# The subspecialty conundrum

**DOI:** 10.4103/0971-3026.69369

**Published:** 2010-08

**Authors:** Rajesh Krishnamurthy

**Affiliations:** E.B. Singleton Department of Pediatric Radiology, Texas Children’s Hospital, Clinical Assistant Professor of Radiology and Pediatrics, Baylor College of Medicine, 6621 Fannin St, MC 2-2521, Houston 77030, Texas, US

Dear Sir,

I read with interest your editorial in the IJRI[1] regarding the status of pediatric radiology training in India. Your statement about the depth of knowledge being more important than the breadth is spot on, as is your warning about radiologists becoming redundant if the specific needs of the clinical subspecialists are not met. I face this reality everyday in my practice.

I did my initial medical training in India, and my radiology, pediatric radiology, and pediatric cardiac MRI subspecialty training in the United States. My practice is almost entirely pediatric cardiovascular imaging and pediatric body MRI. I am fortunate to work in a place where my clinical colleagues practice at the cutting edge of their fields. Hence, the pressure on me as the radiologic provider to stay on top of changes in the field is constant, and the risk of redundancy is real. I welcome this challenge since it allows me the opportunity to be creative and intellectually stimulated at work, and make the occasional original contribution to the literature.

But, I would imagine that my situation would be comparable with only a small proportion of imaging centers in India located in specialty hospitals like children’s hospitals and heart centers. The vast majority of imaging centers in India (and in the United States) are involved in getting the job done, i.e., providing basic radiology services to the masses. In such a situation, breadth of knowledge is king. Indeed, in such a situation, the generalist who performs all kinds of studies at a basic level of competence would be immensely more valuable to the system than the specialist. The cynics among us may say that this is economically driven, and the radiologist reading all types of studies brings in more revenue. But there is an equally strong clinical argument as well.

When we look at the big picture, a group of generalists that provides a constant level of basic, but competent service in all subspecialties will result in better overall care than a random distribution of specialists within a given organization who practice in just their own fields. This is because quality of care is dependent on the system as much as it depends on the individual provider. It is impractical, and in most cases, impossible to create a cadre of subspecialists in each center who will provide constant high-quality service in their selected areas. This will invariably lead to an uneven distribution of quality of care and distribution of resources in that center, and neglect of other specialties. Even major teaching hospitals in the United States have fallen prey to such thinking. Although it may be possible for very large and well-funded organizations to pull this off successfully, there is an inherent inefficiency in such a system that has to be dealt with.

So, one might ask—what is the role of the subspecialist? The ideal scenario is a set-up of generalists in which different radiologists assume the role of a ‘go-to’ expert in each subspecialty. Ideally, such a ‘go-to’ person would be fellowship trained in that area, but abundant experience from any other source will do perfectly well. In such a setting, the ‘go-to’ person will have the following roles: (1) an educator who assumes responsibility for ensuring basic competence of his/her colleagues in that specialty, and keeps them abreast of important changes in the field with continuing education efforts; (2) develops protocols for imaging studies in that specialty and trains colleagues to perform and interpret them appropriately; (3) provides advice on and trouble-shoots technically difficult studies in that field; (4) provides second opinions on clinically challenging cases; and (5) interacts with clinical colleagues at their conferences, and becomes the ‘face’ of the imaging center for the clinicians in that particular specialty. This ensures smooth referrals, and ongoing growth of the referral base. Such a system is more grassroots in its conception, and will handle absences and turnovers better than an elitist model. The responsibility of the departmental head in such a setting is to nurture such talent and provide time and resources for fulfillment of these different specialist roles, so that the ‘go-to’ expert is not unduly stretched fulfilling routine responsibilities. Most large centers across the country already have such a system, albeit in an informal sense.

Another important question that arises in this discussion is the availability of subspecialty training opportunities in India. It is difficult to compare India with the United States when it comes to education in the radiologic subspecialties. Although the practice of radiology in India is quite advanced, system-wide education in radiology is still in its early stages of maturation. Most subspecialty training occurs in private centers or abroad. The new jobs for the trainees are concentrated around the metros, usually in large private hospitals, resulting in an uneven distribution of the talent pool. With rare exceptions, most university- or medical school-based training programs are not equipped with resources for cutting edge subspecialty education in radiology. There is a need for significant restructuring of the system to ensure a more even distribution of specialists across the country.
